# 

**Published:** 1911-09

**Authors:** 


### Date of Issue

Owing to the delay in issuing the August number, prior to the present management, and due to inevitable causes, it has been thought best to equalize the intervals by postponing the publication of the September issue about a week. Hereafter, the Journal will be mailed within the first five days of each month. It seems best to allow some leeway, on account of the occasional advantage in securing an important paper or news item; also, confidentially, because even special practice frequently involves periods of overwork, loss of sleep and out-of-town trips so that, if we laid down an iron clad rule that the Journal must be in the mail of a certain day, it would be necessary to close the forms a week or two earlier than otherwise, in order to run no risk of emergent professional duties.

### BOOKS RECEIVED

A Manual of Pathology and Morbid Anatomy. By T. Henry Green, M.D., F.R.C.P., Consulting Physician to the Charing-Cross Hospital, etc., London. Revised and enlarged by W. Cecil Bosanquet, M.A., M.D., F.R.C.P., Assistant Physician to the Charing-Cross Hospital, etc., London. Large 12mo, 642 pages, with 250 illustrations. Lea & Febiger, Publishers, Philadelphia and New York, 1911. (Cloth, $4.50, net.)

One Hundred Surgical Problems. By James G. Mumford, M.D., Visiting Surgeon to the Massachusetts General Hospital; Instructor in Surgery, Harvard Medical School. Octavo, 354 pages. W. M. Leonard, Boston: 1911. (Cloth, $3.00.)

Manual of the Diseases of the Eye. By Charles H. May, M.D., Chief of Clinic and Instructor in Ophthalmology, College of Physicians and Surgeons, Medical Department, Columbia University. 12mo., 407 pages, with 362 original illustrations. New York: William Wood & Co., 1911. (Cloth, $2.00.)

The Practical Medicine Series. Ten volumes. Under the general editorial charge of Gustavus P. Head, M.D. Vol. IV, Gynecology. Edited by Emilius C. Dudley, A.M., M.D., and C. von Bachellé, M.S , M.D. Vol. V, Obstetrics. Edited by Joseph B. DeLee, A.M., M.D., and Herbert M. Stowes, M.D. Series, 1911. Chicago: The Year Book Publishers. (Prices, $1.25; entire series, $10.00.)

Handbook of Suggestive Therapeutics. By Henry S. Munro, M.D. Third edition, revised and enlarged. 8vo., 409 pages. St. Louis: C. V. Mosby Company. 1911. (Cloth, $4.00.)

Hieronymus Fracastor's Syphilis, from the Original Latin. A translation in prose of Fracastor's immortal poem. Printed on hand-made imported paper; library binding, crown octavo. The Philmar Company, Medical Publishers, Fidelity Building, St. Louis. (Price, $2.00.)

A Manual of Materia Medica. By E. Quin Thornton, M.D., Assistant Professor of Materia Medica in the Jefferson Medical College, Philadelphia. Octavo, 525 pages. Lea & Febiger, Philadelphia and New York, 1911. (Cloth, $3.50, net.)

A Manual of Clinical Diagnosis by Means of Laboratory Methods. By Charles E. Simon, M.D., Professor of Clinical Pathology and Experimental Medicine in the College of Physicians and Surgeons, Baltimore. Seventh edition, enlarged and thoroughly revised. Octavo, 780 pages, with 168 engravings and 25 plates. Lea & Febiger, Philadelphia and New York, 1911. (Cloth, $5.00 net.)

A Manual of Practical Hygiene. By Charles Harrington, M.D., late Professor of Hygiene in the Medical School of Harvard University. Fourth edition, revised and enlarged by Mark W. Richardson, M.D., Secretary to State Board of Health of Massachusetts. Octavo, 850 pages, with 124 engravings and 12 full-page plates, in colors and monochrome. Lea & Febiger, Philadelphia and New York, 1911. (Cloth, $4.50 net.)

